# Diagnostic accuracy of neutrophil-to-lymphocyte ratio, platelet-to-lymphocyte ratio, and neutrophil–lymphocyte-to-platelet ratio biomarkers in predicting bacteremia and sepsis in immunosuppressive patients with cancer: literature review

**DOI:** 10.1097/j.pbj.0000000000000254

**Published:** 2024-06-04

**Authors:** Jose Manuel Martinez, Ana Espírito Santo, Diana Ramada, Filipa Fontes, Rui Medeiros

**Affiliations:** a Oncology Clinical Research Unit IPO Research Center (CI-IPOP), Portuguese Oncology Institute of Porto (IPO Porto)/Porto Comprehensive Cancer Centre (Porto.CCC) & RISE@CI-IPOP (Health Research Network), Porto, Portugal; b Oncology Nursing Research Unit IPO Research Center (CI-IPOP), Portuguese Oncology Institute of Porto (IPO Porto)/Porto Comprehensive Cancer Centre (Porto.CCC) & RISE@CI-IPOP (Health Research Network), Porto, Portugal; c Approach to Precursor Lesions and Early Cancer Research Unit IPO Research Center (CI-IPOP), Portuguese Oncology Institute of Porto (IPO Porto)/Porto Comprehensive Cancer Centre (Porto.CCC) & RISE@CI-IPOP (Health Research Network), Porto, Portugal; d Public Health Department and Forensic Sciences and Medical Education, Faculty of Medicine, University of Porto, Porto, Portugal; e Molecular Oncology and Viral Pathology Group Research Unit IPO Research Center (CI-IPOP), Portuguese Oncology Institute of Porto (IPO Porto)/Porto Comprehensive Cancer Centre (Porto.CCC) & RISE@CI-IPOP (Health Research Network), Porto, Portugal

**Keywords:** sepsis, immunosuppression, bacteremia, biomarker, oncology

## Abstract

**Background:**

This literature review explores the role of neutrophil-to-lymphocyte ratio (NLR), platelet-to-lymphocyte ratio (PLR), and neutrophil–lymphocyte-to-platelet ratio (NLPR) biomarkers, as potential indicators for predicting bacteremia and sepsis in patients with cancer.

**Objective:**

Tracing the evolution of interest in this area since 2001, the aim of this review was to report a comprehensive overview of current knowledge and gaps, particularly in patients undergoing immunosuppression.

**Summary of Findings:**

The literature research indicates the potential of NLR, PLR, and other biomarkers in diagnosing and predicting sepsis, with some studies emphasizing their value in mortality prediction. A specific focus on bacteremia shows the effectiveness of NLR and PLR as early indicators and prognostic tools, though mostly in noncancer patient populations. While NLR and PLR are promising in general cancer patient populations, the review addresses the challenges in applying these biomarkers to patients with neutropenic and lymphopenic cancer. The NLPR could be considered a significant biomarker for inflammation and mortality risk in various medical conditions, yet its diagnostic accuracy in patients with immunosuppressed cancer is not extensively validated.

**Conclusion:**

This review offers a snapshot of the current research on biomarkers in patients with immunocompromised cancer in the sepsis and bacteremia area. More focused research on their application is necessary. This gap underscores an opportunity for future studies to enhance diagnostic and prognostic capabilities in this high-risk group.

## Introduction

**Figure 1.**
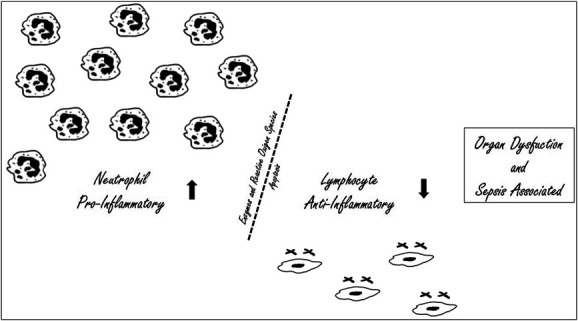
Imbalance between the pro-inflammatory and the anti-inflammatory response, neutrophils/lymphocytes.

The role of biomarkers as predictors of bacteremia and sepsis has been the subject of several studies in the last two decades. Neutrophil–lymphocyte ratio (NLR) is calculated as the neutrophil count divided by the lymphocyte count, associated with the imbalance between neutrophils and lymphocytes in the pro-inflammatory and anti-inflammatory response (Fig. 1). This ratio has been considered an important biomarker predictor of cancer prognosis, for the occurrence of sepsis, and the decision of the therapeutical regimen.^1,2^

**Figure 2.**
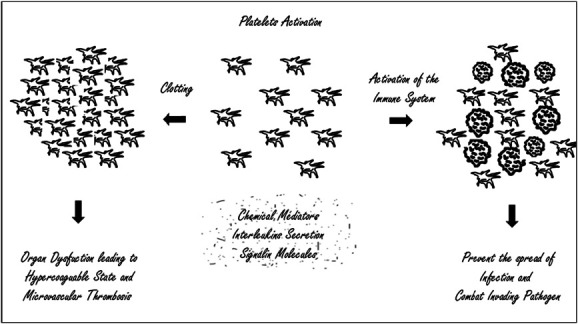
Imbalance between coagulation and immune system response.

In recent years, new insights into NLR have emerged, either in isolation or in correlation with other ratios and hemogram parameters, such as the platelet–lymphocyte ratio (PLR), which reflects the correlation between coagulation and immune systems in septic patients (Fig. 2).^1-8^ On the other hand, the neutrophil–lymphocyte-to-platelet ratio (NLPR) is increasingly used as an accuracy parameter to assess the degree of inflammation. Both biomarkers are frequently associated with a poorer prognosis and elevated mortality rates in critically ill patients. However, although literature exists regarding these biomarkers, for diagnosis and prognosis of sepsis and bacteremia in the general population, especially in emergency departments, their use has not been extensively validated among patients with cancer.^1-8^

The oncology patient's journey is often characterized by multiple hospitalizations, diagnostic tests, and several complications, particularly febrile neutropenia in immunocompromised patients.^9-11^ The neutropenia can significantly impact bacterial load and host–microbe interactions, leading to altered immune response, changes in microbial composition, and possibly dysbiosis, which can impair responses to antibiotic and corticosteroid treatments.^12-14^ In cases where sepsis is suspected, a blood culture test remains the gold standard for confirming bacteremia.^15^ However, the sensitivity and specificity of the blood culture testing may be influenced by several variables, including the timing of specimen collection, the volume of blood sampled, prior antibiotic use, the presence of fastidious or slow-growing organisms, adequacy of culture media, and the incubation period of the culture.^16^ Consequently, the reliability of blood culture tests in accurately detecting the presence or absence of infection may be compromised, increasing the risk of false-negative or false-positive results, potentially leading to incorrect or delayed diagnoses and inappropriate treatments.^13^

In the context of bacteremia and sepsis, there is growing attention on studies investigating blood culture assessment and the types of microorganisms involved.^15^ However, scientific knowledge remains limited, particularly concerning patients with cancer undergoing neutropenia and lymphopenia.^17-19^ Therefore, the aim of this review was to focus on the state-of-the-art diagnostic accuracy of NLR, PLR, and NLPR in predicting bacteremia and sepsis in immunosuppressed cancer patients.

## Results/Discussion

There is an increasing interest in the relationship between hematological biomarkers and various medical conditions, particularly in oncology and infectious diseases, aiming to elucidate their diagnostic and prognostic significance comprehensively. This discussion is structured into five subchapters, each focusing on a specific aspect of this complex interplay. This structured approach not only facilitates a systematic exploration of the topics but also underscores the multifaceted roles of NLR, PLR, and NLPR across different medical disciplines (Table 1).

**Table 1:** Multifaceted roles of NLR, PLR, and NLPR across different medical disciplines: a representative selection of the literature review.

First author	Country (Y)	Clinical context	Sex, male, n (%)	Age, y*	Biomarkers under study†	Main findings
Tang^15^	China 2020	Patients with suspected infection who had blood culture sampling (n = 825)	Positive BC group: 360 (52.10) Negative BC group: 67 (50)	Positive BC group: 61 (46–72) Negative BC group: 53 (28–69)	PLR, P-LCC, LYM, PLT, NLR, MVP, MVP/PLT	Hematological parameters may improve the ability to differentiate between patients with bloodstream infections caused by a different range of pathogens
Jager^17^	Netherlands 2010	Adult patients admitted to the emergency department with suspected community-acquired bacteremia (n = 746)	44 (47.8)	66 (18–96)	Lymphocyte, NLCR	Lymphocytopenia and NLCR are better predictors of bacteremia than routine parameters such as CRP level, WBC count, and neutrophil count
Qiu^19^	China 2024	Sepsis patients with lymphopenia (n = 172)	130 (75.58)	57.57 ± 25.01	NLR, PLR	NLR and PLR independently predict hospital mortality in sepsis patients with lymphopenia
Hwang^20^	South Korea 2017	Patients with sepsis or septic shock admitted to the emergency department (n = 1395)	787 (56.4)	65 (55–73)	NLR	NLR is independently linked to 28-day mortality in patients with severe sepsis and septic shock
Liu^21^	China 2016	Patients with sepsis admitted to the ICU (n = 333)	188 (56.46)	70.26 ± 15.79	NLR	Increased NLR levels were independently associated with unfavorable clinical prognosis in patients with sepsis
Ljungstrom^22^	Sweden 2017	Patients with sepsis admitted to the emergency department (n = 1572)	875 (55.7)	71 (58–81)	NLCR, CRP, PCT	Combinations of biomarkers have shown the potential to enhance the diagnosis of confirmed bacterial sepsis in the most critically ill patients. However, in less severe septic conditions, either NLCR or PCT alone may exhibit equivalent performance
Kaushik^23^	India 2018	Patients with sepsis (n = 56)	23 (41)	30.0 ± 28.0	NLR	NLR can be a useful diagnostic and prognostic marker in sepsis
Lorente^24^	Spain 2020	Patients with sepsis (n = 203)	140 (69)	60 (49–70)	NLR	Association between NLR in the first seven days of sepsis and mortality
Terradas^25^	Spain 2012	Patients with a first episode of community-acquired or healthcare-related bacteremia during hospital admission	1316 (56.95)	67.70 ± 16.26	NLCR, Eosinophil count	Eosinopenia and persistence of an NLCR were independent markers of mortality in patients with bacteremia
Laukemann^26^	Switzerland 2015	Patients with suspected infection who had blood culture sampling admitted to the emergency department (n = 1083)	623 (57.6)	67 (53–78)	PCT, NLCR, CRP, RDW	PCT and NLCR effectively anticipate bacteremia, thereby reducing the need for unnecessary blood cultures
Zhang^27^	China 2016	Patients with suspected infection who had blood culture sampling (n = 120)	Positive BC group: 33 (55) Negative BC group: 40 (66.7)	Positive BC group: 62.6 ± 14.9 Negative BC group: 66 ± 15.3	RDW, PDW, NLCR, PCT, CRP	NLCR is effective for predicting sepsis
Djordjevic^28^	Serbia 2018	Adult critically ill and injured patients, admitted to the surgical intensive care unit (n = 392)	236 (60.2)	53.67 ± 18.26	NLR, MLR, PLR, and MPV/PC	The nature of bacteremia influences MPV/PC, MLR, and PLR
Yang^29^	China 2022	Cancer patients with sepsis (n = 317)	172 (75.1)	63.5 ± 10	NLR, BNP	A nomogram incorporating NLR d3, BNP d3, 72-hour fluid accumulation and SOFA score accurately predict 28-day prognosis in cancer patients with sepsis in the ICU

* Disclaimer: differences in the age reporting across the studies were observed (mean ± standard deviation or median [min–max]), direct comparisons might be limited or should be made with caution.

† BNP, B-Type Natriuretic Peptide; CRP, C-Reactive Protein; LYM, combinations of lymphocyte count; MDW, Monocyte Distribution Width; MPV/PC, Mean Platelet Volume-to-Platelet Count; N/LPR, Neutrophil-to-Lymphocyte/Platelet Ratio; NLCR, Neutrophil-to-Lymphocyte Count Ratio; NLR, Neutrophil-to-Lymphocyte Ratio; PCT, Procalcitonin; PDW, Platelet Distribution Width; PDW/PC, Platelet Distribution Width-to-Platelet Count; P-LCC, platelet larger cell count; PLR, Platelet-to-Lymphocyte Ratio; PNR, Platelet-to-Neutrophil Ratio; RDW, Red Cell Distribution Width; RDW/PC, Red Cell Distribution Width-to-Platelet Count.

### Chapter 1: variability in neutrophil, lymphocyte, and platelet ratios: the impact of hematological disorders and oncological therapies

In the context of cancer, NLR and PLR have emerged as significant biomarkers of cancer-related details and valid prognostic indicators, particularly for solid tumors.^30,31^ Several studies have identified specific NLR cutoff values depending on the study field,^1–3,7,32,33^ with values above a certain threshold (usually around 3.0) associated with worse prognosis across different cancers.^1-3^ On the other hand, low values below 0.7 are associated with morbidity and mortality in critically ill septic patients.^20^

The NLR can be used for cancer stratification, correlating with tumor size, stage, metastatic potential, and lymphatic invasion. Furthermore, it plays an independent prognostic role in cancer-free survival and is valuable for monitoring oncological therapy outcomes. Elevated PLR is negatively associated with overall survival and serves as a predictive marker for neoadjuvant chemotherapy effectiveness in solid tumors. NLPR is increasingly recognized as a significant biomarker in various fields such as COVID-19, identification of causative pathogens, multiple myeloma, small-cell lung cancer, and burn patients, particularly for prognosis and risk assessment.^2,15,21,22^

Inflammatory responses, infections, bone marrow invasion, cancer treatments, and hematological or lymphatic system disorders can influence these blood count ratios, leading to NLR variations and conflicting research results on diagnostic accuracy for bacteremia.^5,6,22^

Hematology disorders significantly impact blood studies, but solid tumors may also induce a systemic inflammatory response that may release cytokines and growth factors stimulating neutrophil production in the bone marrow.^7,34,35^ Hematological disorders exhibit nondiscriminatory prevalence across diverse conditions. The bone marrow could be affected and disrupted with normal blood cell production in various clinical scenarios, affecting individuals regardless of their health status, age, gender, or other demographic factors.^36^ Immunocompromised patients undergoing chemotherapy and radiation treatments may experience a decrease in neutrophils, platelets, or lymphocytes compromising host immune response and increasing susceptibility to bacterial infections.^37^ Systematic inflammatory responses, lymphocytopenia, and thrombocytosis interact with neutrophilia in varying levels of these ratios. Moreover, cancer treatments may induce severe neutropenia, lymphocytopenia, and thrombocytopenia, further affecting these ratios.

### Chapter 2: biomarkers in sepsis and bacteremia: enhancing diagnostic accuracy and prognostic prediction

The literature has several studies focusing on the diagnosis and prediction of sepsis and bacteremia, offering diverse perspectives, particularly in noncancer patient populations. Gurol et al^33^ (2015) identified an NLR ≥5 as a potential marker for bacterial infection. Ljungström et al^22^ (2017) reported that combining biomarkers (PCT, NLR, CRP) improve the diagnostic accuracy for bacterial sepsis, regardless of using Sepsis-2 or Sepsis-3 criteria. Expanding on the temporal aspects, Kaushik et al^23^ (2018) noted that elevated NLR levels are observed in the early phase of sepsis, aiding diagnosis, while late-phase values serve as prognostic indicators. Further supporting the prognostic value of NLR, Shi et al^31^ (2019) found that a day 5 NLPR and NLR were independently correlated with in-hospital mortality.

Shen et al^30^ (2019) studied PLR as a prognostic predictor of mortality in sepsis, involving 5537 patients with sepsis, of varied underlying conditions, and observed that PLR values at admission were associated with increased mortality risk. Complementing these findings, Yoldas et al (2020) evaluated NLR and PLR as markers for predicting hospital stay and mortality in intensive care settings, with a focus on noncancer patients. They found that the median NLR was 2.06 (range: 1.18–21.68) for survivors and 10.42 (range: 2.85–48.20) for deceased patients. Similarly, the median PLR in the deceased group was significantly higher than that in the survivor group [268.9 (range: 150–3000) and 55.7 (range: 11.8–152.5), respectively]. These markers, along with C-reactive protein levels, were identified as predictors of mortality in critically ill adult patients.^38^

Building on this approach to enhance diagnostic sensitivity, Spoto et al^39^ (2021) studied the association between clinical signs and biomarkers to increase diagnostic sensitivity of sepsis and predict disease severity and mortality, finding that NLR and PLR are associated with 90-day mortality in patients with sepsis. Lorente et al^24^ (2022) evaluate the association between NLR in the first seven days of sepsis and mortality, controlling for sepsis severity. In addition, Botos et al^32^ (2023) identified day 14 of NLR, low peripheral blood-derived NLR (dNLR), and PLR as independent predictors of septic shock and mortality in patients with sepsis.

### Chapter 3: evaluating NLR and PLR as predictive tools in bacteremia conditions

The exploration of biomarkers, in patients with bacteremia and blood culture testing, provides valuable insights applicable across diverse clinical contexts.

Terradas et al^25^ (2012) conducted a retrospective analysis focusing on patients experiencing their first episode of community-acquired or healthcare-related bacteremia during hospital admission. This approach allowed for the examination of eosinophil count and NLR in a specific clinical context, revealing their prognostic value. This study excluded patients with hematological malignancies and identified sustained eosinopenia and NLR >7 as independent markers of mortality in patients with bacteremia. Expanding on this concept, Lowsby et al^40^ (2015) studied NLR as an early indicator of bloodstream infection in the emergency department, suggesting its potential for guiding the clinical management of suspected bacteremia cases. Similarly, Loonen et al (2014) and Laukemann et al (2015) studied clinical scores incorporating biomarkers and biomarker levels to predict bacteremia and reduce unnecessary blood cultures, with NLR providing effectively for sepsis prediction.^26,41^

Shifting focus to a broader patient population, Salciccioli et al^42^ (2015) studied 5056 critically ill adult patients, revealing that an elevated NLR at ICU admission is associated with higher 28-day mortality, particularly in nonseptic patients. The study suggests that NLR may be a useful indicator of inflammatory response and prognosis in critical illness but highlights the need for more research to fully understand these associations. Hwang et al^20^ (2016) conducted a retrospective cohort study with 1395 emergency department patients, categorizing them by initial NLR values and identifying persistent low or high NLR as significant risk factors for 28-day mortality, particularly highlighting the potential utility of NLR among patients with cancer for management of septic complications.

Taking a prognostic perspective, Zhang et al^27^ (2016) studied NLR according to blood culture test results, excluding patients with hematological malignancies and bone marrow infiltrations, and found high diagnostic efficiency of NLR predicting sepsis. Similarly, Djorjdjevic et al^28^ (2018) studied a prospective cohort of 392 critically ill adult and injured patients, observing NLR and PLR with different blood cultures and concluding NLR as an independent predictor of mortality. Focusing on platelet count as a biomarker, Sinha et al^43^ (2021) studied a critically ill population of 130 adult patients, analyzing NLR, PLR, and mean corpuscular volume (MCV) with 28-day hospital mortality and length of stay outcomes. The study identified thrombocytopenia on day 1 and decreased eosinophil count on day 3 as predictors of 28-day mortality in patients with sepsis.

### Chapter 4: advancing diagnostic precision: biomarker efficacy in bacteremia and sepsis beyond cancer contexts

Moving from broader cancer-related studies to specific research, Jager et al^17^ (2010) conducted an insightful investigation into the diagnostic capabilities of lymphocytopenia and NLR in bacteremia. This study evaluated the effectiveness of these biomarkers compared with conventional biomarkers in predicting bacteremia among patients presenting to the emergency department with positive blood cultures. The study population included 184 patients (92 patients with confirmed bacteremia and a control group of 92 patients without bacteremia). Notably, patients with hematological diseases, those receiving chemotherapy and those on glucocorticoids, were excluded. The results showed that lymphocytopenia and NLR are superior predictors of bacteremia compared with traditional markers, such as C-reactive protein and white blood cell count. NLR, in particular, exhibited higher sensitivity and specificity, highlighting its potential as a diagnostic tool in emergency medicine. Building upon these findings, Naess et al^18^ (2017) conducted a study focusing on the roles of NLR and monocyte-to-lymphocyte ratio (MLR) in patients with fever of unknown origin. The study involved 299 patients and analyzed NLR and MLR with final diagnostic groups and fever duration. Higher NLR and MLR values were associated with a higher probability of bacterial infection and a lower probability of viral infection. The study included a subset of 26 patients with compromised immune systems, primarily transplant recipients and patients with HIV, excluding patients with leukemia due to the potential impacts on white blood cell counts.

From an alternative perspective, Qiu et al^19^ (2024) conducted a retrospective analysis of 172 sepsis patients with lymphopenia to assess the prognostic value of NLR and PLR. The study identified cutoff values of 18.93 for NLR and 377.50 for PLR in predicting hospital mortality. Higher NLR and PLR values were found to be independent predictors of mortality, providing valuable insights for clinical decision making in sepsis management.

### Chapter 5: emerging research and future directions of cytopenic hematological biomarkers in patients with cancer: insights and unmet needs

NLR and PLR have emerged as significant prognostic indicators in recent studies, due to their accessibility and quantification. In 2020, Joshua Farkas proposed that NLR and the fraction of immune granulocytes warrant further investigations as emergency indicators.^7^ Similarly, a prospective study by Kim et al^44^ (2019) emphasized the importance of PLR following granulocyte colony–stimulating factor (G-CSF) administration in patients with septic shock. This study found that PLR combined with the APACHE II score, independently predicts one-month survival in these patients.

Zahorec^2^ (2021) in the review “Neutrophil-to-Lymphocyte Ratio: Past, Present, and Future,” highlighted the extensive use of NLR to evaluate inflammatory responses and immune system activation across various medical conditions, including cancer. During the COVID pandemic, a retrospective study led by Yang et al^29^ (2022) in China emphasized the predictive role of NLR in cancer patients with sepsis. Key variables such as NLR, brain natriuretic peptide (BNP), fluid accumulation, and the Sequential Organ Failure Assessment (SOFA) score were identified as independent risk factors of 28-day mortality. A developed nomogram incorporating these factors demonstrated good predictive accuracy. Up to this date, based on our current knowledge, this article is one of a few studies conducted in a cancer facility. Therefore, the gap in the literature for immunosuppressive patients becomes evident when considering the NLR's role in typical oncological settings. Comprehensive reviews by Buonacera et al^8^ (2022) and Firment et al^1^ (2024) highlighted the applicability and effectiveness of the NLR and the Zahorec index (a biomarker derived by the ratio between neutrophil count and lymphocyte count) across various clinical conditions, including sepsis, cancer, and postsurgical responses. The review by Buonacera et al^8^ (2022) explores how chronic inflammation contributes to tumorigenesis and cancer progression, emphasizing NLR's significance as a biomarker for ongoing cancer-related inflammation, and NLR correlates with tumor size, stage, metastatic potential, and lymphatic invasion, making it a valuable tool for stratifying patients with cancer and predicting outcomes. While hematological biomarkers have demonstrated utility in standard cancer patient populations, gaps exist regarding their application in patients with neutropenic and lymphopenic cancer which requires further investigation in typical oncology settings.

### The dual nature of current biomarker research: successes and limitations

There are some gaps in the scientific research. First, there is a notable lack of focus on immunocompromised patients with cancer. Despite this limitation, several studies provide valuable insights into the general applicability of NLR, PLR, and NLPR as biomarkers, setting a foundation for future research specifically tailored to immunocompromised patients. Second, studies often face potential confounding factors of diverse natures. Highlighting these potential confounders is crucial for guiding future research to control for these variables, thereby enhancing the understanding of biomarkers in specific patient populations. Third, caution is needed in generalization findings. A cautious approach to generalization underscores the need for more specific research, compelling the scientific community to address these gaps.

Conversely, there has been a marked and successful surge in interest in this field, providing a snapshot of the current state of research and identifying trends and gaps crucial for guiding future research directions.

## Conclusion

In conclusion, while the literature review demonstrates the utility of hematological biomarkers in standard cancer patient populations, particularly in predicting mortality outcomes, it does not specifically address their application in patients with neutropenic and lymphopenic cancer. This emphasized the need for more dedicated studies in this domain.

## References

[1] Firment J Hulin I. Zahorec index or Neutrophil-to-lymphocyte ratio, valid biomarker of inflammation and immune response to infection, cancer and surgery. Bratisl Lek Listy. 2024;125:75–83. 38219059 10.4149/BLL_2024_012

[2] Zahorec R. Neutrophil-to-lymphocyte ratio, past, present and future perspectives. Bratisl Lek Listy. 2021;122:474–88. 34161115 10.4149/BLL_2021_078

[3] Huang Z Fu Z Huang W Huang K. Prognostic value of neutrophil-to-lymphocyte ratio in sepsis: a meta-analysis. Am J Emerg Med. 2020;38:641–7. 31785981 10.1016/j.ajem.2019.10.023

[4] Orfanu AE Popescu C Leuștean A The importance of haemogram parameters in the diagnosis and prognosis of septic patients. J Crit Care Med (Targu Mures). 2017;3:105–10. 29967880 10.1515/jccm-2017-0019 PMC5769899

[5] Jiang J Liu R Yu X . The neutrophil-lymphocyte count ratio as a diagnostic marker for bacteraemia: a systematic review and meta-analysis. Am J Emerg Med. 2019;37:1482–9. 30413366 10.1016/j.ajem.2018.10.057

[6] Russell CD Parajuli A Gale HJ . The utility of peripheral blood leucocyte ratios as biomarkers in infectious diseases: a systematic review and meta-analysis. J Infect. 2019;78:339–48. 30802469 10.1016/j.jinf.2019.02.006 PMC7173077

[7] Farkas JD. The complete blood count to diagnose septic shock. J Thorac Dis. 2020;12(Suppl 1):S16–21. 32148922 10.21037/jtd.2019.12.63 PMC7024748

[8] Buonacera A Stancanelli B Colaci M Malatino L. Neutrophil to lymphocyte ratio: an emerging marker of the relationships between the immune system and diseases. Int J Mol Sci. 2022;23:3636. 35408994 10.3390/ijms23073636 PMC8998851

[9] Boccia R Glaspy J Crawford J Aapro M. Chemotherapy-induced neutropenia and febrile neutropenia in the US: a beast of burden that needs to be tamed? Oncologist. 2022;27:625–36. 35552754 10.1093/oncolo/oyac074 PMC9355811

[10] Peseski AM McClean M Green SD Beeler C Konig H. Management of fever and neutropenia in the adult patient with acute myeloid leukemia. Expert Rev Anti Infect Ther. 2021;19:359–78. 32892669 10.1080/14787210.2020.1820863

[11] Aagaard T Reekie J Jørgensen M . Mortality and admission to intensive care units after febrile neutropenia in patients with cancer. Cancer Med. 2020;9:3033–42. 32144897 10.1002/cam4.2955 PMC7196064

[12] Martínez JA Pozo L Almela M . Microbial and clinical determinants of time-to-positivity in patients with bacteraemia. Clin Microbiol Infect. 2007;13:709–16. 17484763 10.1111/j.1469-0691.2007.01736.x

[13] Zhang Q Li D Bai C . Clinical prognostic factors for time to positivity in cancer patients with bloodstream infections. Infection. 2016;44:583–8. 27084368 10.1007/s15010-016-0890-2

[14] Shah T Baloch Z Shah Z Cui X Xia X. The intestinal microbiota: impacts of antibiotics therapy, colonization resistance, and diseases. Int J Mol Sci. 2021;22:6597. 34202945 10.3390/ijms22126597 PMC8235228

[15] Tang W Zhang W Li X . Hematological parameters in patients with bloodstream infection: a retrospective observational study. J Infect Dev Ctries. 2020;14:1264–73. 33296339 10.3855/jidc.12811

[16] Passerini R Riggio D Radice D . Interference of antibiotic therapy on blood cultures time-to-positivity: analysis of a 5-year experience in an oncological hospital. Eur J Clin Microbiol Infect Dis. 2009;28:95–8. 18663498 10.1007/s10096-008-0594-3

[17] de Jager CP van Wijk PT Mathoera RB de Jongh-Leuvenink J van der Poll T Wever PC. Lymphocytopenia and neutrophil-lymphocyte count ratio predict bacteremia better than conventional infection markers in an emergency care unit. Crit Care. 2010;14:R192. 21034463 10.1186/cc9309 PMC3219299

[18] Naess A Nilssen SS Mo R Eide GE Sjursen H. Role of neutrophil to lymphocyte and monocyte to lymphocyte ratios in the diagnosis of bacterial infection in patients with fever. Infection. 2017;45:299–307. 27995553 10.1007/s15010-016-0972-1 PMC5488068

[19] Qiu X Wang Q Zhang Y Zhao Q Jiang Z Zhou L. Prognostic value of neutrophils-to-lymphocytes ratio and platelets-to-lymphocytes ratio in sepsis patients with lymphopenia. Biomark Insights. 2024;19:11772719231223156. 38186669 10.1177/11772719231223156 PMC10768602

[20] Hwang SY Shin TG Jo IJ . Neutrophil-to-lymphocyte ratio as a prognostic marker in critically-ill septic patients. Am J Emerg Med. 2017;35:234–9. 27806894 10.1016/j.ajem.2016.10.055

[21] Liu X Shen Y Wang H Ge Q Fei A Pan S. Prognostic significance of neutrophil-to-lymphocyte ratio in patients with sepsis: a prospective observational study. Mediators Inflamm. 2016;2016:8191254. 27110067 10.1155/2016/8191254 PMC4823514

[22] Ljungström L Pernestig AK Jacobsson G Andersson R Usener B Tilevik D. Diagnostic accuracy of procalcitonin, neutrophil-lymphocyte count ratio, C-reactive protein, and lactate in patients with suspected bacterial sepsis. PLoS One. 2017;12:e0181704. 28727802 10.1371/journal.pone.0181704 PMC5519182

[23] Kaushik R Gupta M Sharma M . Diagnostic and prognostic role of neutrophil-to-lymphocyte ratio in early and late phase of sepsis. Indian J Crit Care Med. 2018;22:660–3. 30294133 10.4103/ijccm.IJCCM_59_18 PMC6161585

[24] Lorente L Martín MM Ortiz-López R . Association between neutrophil-to-lymphocyte ratio in the first seven days of sepsis and mortality. Enferm Infecc Microbiol Clin. 2022;40:235–40. 10.1016/j.eimce.2020.11.022 35577441

[25] Terradas R Grau S Blanch J . Eosinophil count and neutrophil-lymphocyte count ratio as prognostic markers in patients with bacteremia: a retrospective cohort study. PLoS One. 2012;7:e42860. 22912753 10.1371/journal.pone.0042860 PMC3415420

[26] Laukemann S Kasper N Kulkarni P . Can we reduce negative blood cultures with clinical scores and blood markers? Results from an observational cohort study. Medicine (Baltimore). 2015;94:e2264. 26656373 10.1097/MD.0000000000002264 PMC5008518

[27] Zhang HB Chen J Lan QF Ma XJ Zhang SY. Diagnostic values of red cell distribution width, platelet distribution width and neutrophil-lymphocyte count ratio for sepsis. Exp Ther Med. 2016;12:2215–9. 27698714 10.3892/etm.2016.3583 PMC5038364

[28] Djordjevic D Rondovic G Surbatovic M . Neutrophil-to-Lymphocyte ratio, monocyte-to-lymphocyte ratio, platelet-to-lymphocyte ratio, and mean platelet volume-to-platelet count ratio as biomarkers in critically ill and injured patients: which ratio to choose to predict outcome and nature of bacteremia? Mediators Inflamm. 2018;2018:3758068. 30116146 10.1155/2018/3758068 PMC6079471

[29] Yang Y Dong J Li Y . Development and validation of a nomogram for predicting the prognosis in cancer patients with sepsis. Cancer Med. 2022;11:2345–55. 35182022 10.1002/cam4.4618 PMC9189475

[30] Shen Y Huang X Zhang W. Platelet-to-lymphocyte ratio as a prognostic predictor of mortality for sepsis: interaction effect with disease severity-a retrospective study. BMJ Open. 2019;9:e022896. 10.1136/bmjopen-2018-022896 PMC6352809 30782690

[31] Shi Y Yang C Chen L Cheng M Xie W. Predictive value of neutrophil-to-lymphocyte and platelet ratio in in-hospital mortality in septic patients. Heliyon. 2022;8:e11498. 36439769 10.1016/j.heliyon.2022.e11498 PMC9681647

[32] Botoş ID Pantiş C Bodolea C . The dynamics of the neutrophil-to-lymphocyte and platelet-to-lymphocyte ratios predict progression to septic shock and death in patients with prolonged intensive care unit stay. Medicina (Kaunas). 2022;59:32. 36676656 10.3390/medicina59010032 PMC9861709

[33] Gürol G Çiftci İ H Terzi HA Atasoy AR Ozbek A Köroğlu M. Are there standardized cutoff values for neutrophil-lymphocyte ratios in bacteremia or sepsis? J Microbiol Biotechnol. 2015;25:521–5. 25341467 10.4014/jmb.1408.08060

[34] van Rensburg J Davids S Smuts C Davison GM. Use of full blood count parameters and haematology cell ratios in screening for sepsis in South Africa. Afr J Lab Med. 2023;12:2104. 37151816 10.4102/ajlm.v12i1.2104 PMC10157447

[35] Agnello L Giglio RV Bivona G . The value of a complete blood count (CBC) for sepsis diagnosis and prognosis. Diagnostics (Basel). 2021;11:1881. 34679578 10.3390/diagnostics11101881 PMC8534992

[36] Bennett JE Dolin R Blaser MJ. Mandell, Douglas, and Bennett’s Principles and Practice of Infectious Diseases. 8th ed. Philadelphia, PA: Elsevier; 2015.

[37] Varon J. Hematologic Disorders. Handbook of Critical and Intensive Care Medicine. Cham: Springer; 2016:159–80.

[38] Yoldas H Karagoz I Ogun MN . Novel mortality markers for critically ill patients. J Intensive Care Med. 2020;35:383–5. 29334832 10.1177/0885066617753389

[39] Spoto S Lupoi DM Valeriani E . Diagnostic accuracy and prognostic value of neutrophil-to-lymphocyte and platelet-to-lymphocyte ratios in septic patients outside the intensive care unit. Medicina (Kaunas). 2021;57:811. 34441017 10.3390/medicina57080811 PMC8399559

[40] Lowsby R Gomes C Jarman I . Neutrophil to lymphocyte count ratio as an early indicator of blood stream infection in the emergency department. Emerg Med J. 2015;32:531–4. 25183249 10.1136/emermed-2014-204071

[41] Loonen AJ de Jager CP Tosserams J . Biomarkers and molecular analysis to improve bloodstream infection diagnostics in an emergency care unit. PLoS One. 2014;9:e87315. 24475269 10.1371/journal.pone.0087315 PMC3903623

[42] Salciccioli JD Marshall DC Pimentel MA . The association between the neutrophil-to-lymphocyte ratio and mortality in critical illness: an observational cohort study. Crit Care. 2015;19:13. 25598149 10.1186/s13054-014-0731-6 PMC4344736

[43] Sinha H Maitra S Anand RK . Epidemiology and prognostic utility of cellular components of hematological system in sepsis. Indian J Crit Care Med. 2021;25:660–7. 34316146 10.5005/jp-journals-10071-23874 PMC8286394

[44] Kim YJ Kang J Ryoo SM Ahn S Huh JW Kim WY. Platelet-lymphocyte ratio after granulocyte colony stimulating factor administration: an early prognostic marker in septic shock patients with chemotherapy-induced febrile neutropenia. Shock. 2019;52:160–5. 30148761 10.1097/SHK.0000000000001256

